# Insights into Emerging Begomovirus–Deltasatellite Complex Diversity: The First Deltasatellite Infecting Legumes

**DOI:** 10.3390/biology10111125

**Published:** 2021-11-02

**Authors:** Elvira Fiallo-Olivé, Liseth Bastidas, Dorys T. Chirinos, Jesús Navas-Castillo

**Affiliations:** 1 Instituto de Hortofruticultura Subtropical y Mediterránea “La Mayora” (IHSM-UMA-CSIC), Consejo Superior de Investigaciones Científicas, Avenida Dr. Wienberg s/n, 29750 Algarrobo-Costa, Málaga, Spain; jnavas@eelm.csic.es; 2 Departamento Fitosanitario, Facultad de Agronomía, Universidad del Zulia, Maracaibo 4005, Zulia, Venezuela; liseth.bastidas@gmail.com; 3 Facultad de Ingeniería Agronómica, Universidad Técnica de Manabí, Portoviejo 130105, Manabí, Ecuador; dorys.chirinos@utm.edu.ec

**Keywords:** *Geminiviridae*, begomoviruses, deltasatellites, legumes, black gram, common bean, cowpea, faba bean, mung bean

## Abstract

**Simple Summary:**

Legumes play an important nutritional role in the diets of millions of people, mainly in developing countries, but their productivity is seriously affected by a variety of pathogens including viruses. In the last few decades, a number of whitefly-transmitted viruses have emerged mainly in tropical and subtropical areas worldwide including the DNA-containing begomoviruses. A survey of leguminous plants for the presence of begomoviruses was conducted in Venezuela, an understudied country in this regard, even when a variety of symptoms resembling those caused by begomoviruses has been observed in leguminous crops for 20 years. As a result, begomoviruses belonging to four novel species have been discovered and molecularly characterized. In addition, a novel deltasatellite, a small DNA molecule associated with some begomoviruses, has been found to be associated with cabbage leaf curl virus. This is the first time that a deltasatellite has been found to infect legumes. Our results illustrate the increasing complexity faced by researchers and breeders looking to develop control strategies against these emerging pathogens.

**Abstract:**

Begomoviruses and associated DNA satellites are involved in pathosystems that include many cultivated and wild dicot plants and the whitefly vector *Bemisia tabaci*. A survey of leguminous plants, both crops and wild species, was conducted in Venezuela, an understudied country, to determine the presence of begomoviruses. Molecular analysis identified the presence of bipartite begomoviruses in 37% of the collected plants. Four of the six begomoviruses identified constituted novel species, and two others had not been previously reported in Venezuela. In addition, a novel deltasatellite (cabbage leaf curl deltasatellite, CabLCD) was found to be associated with cabbage leaf curl virus (CabLCV) in several plant species. CabLCD was the first deltasatellite found to infect legumes and the first found in the New World to infect a crop plant. Agroinoculation experiments using *Nicotiana benthamiana* plants and infectious viral clones confirmed that CabLCV acts as a helper virus for CabLCD. The begomovirus–deltasatellite complex described here is also present in wild legume plants, suggesting the possible role of these plants in the emergence and establishment of begomoviral diseases in the main legume crops in the region. Pathological knowledge of these begomovirus–deltasatellite complexes is fundamental to develop control methods to protect leguminous crops from the diseases they cause.

## 1. Introduction

Legumes are grown globally for human consumption, playing an important nutritional role in the diets of millions of people, mainly in developing countries. Additionally, they are used for livestock feeding and, in some cases, as soil-enhancing green manure. Legumes belong to the family Fabaceae (syn. Leguminosae), one of the largest angiosperm plant families with more than 750 genera and about 19,000 known species [1]. Legume production is constrained by many pests and diseases, especially in the tropics and subtropics [2,3,4].

Begomoviruses (genus *Begomovirus*, family *Geminiviridae*) are circular single-stranded DNA plant viruses with twin (geminate) quasi-icosahedral virions transmitted in nature by whiteflies (Hemiptera: Aleyrodidae) of the *Bemisia tabaci* complex. Begomoviruses are clustered in four major phylogenetic groups: New World, Old World, sweepoviruses, and legumoviruses. Begomovirus genomes can either be bipartite or monopartite. Most New World begomoviruses have a bipartite genome that consists of DNA-A and DNA-B components, each being 2.5–2.6 kb. Both genome components share approximately 200 nucleotides within the intergenic region, which contains a stem-loop structure with the highly conserved nonanucleotide (TAATATTAC) found at the origin of replication. DNA-A encodes the coat protein (and pre-coat protein in Old World begomoviruses) in the viral sense, and the replication-associated protein, a transcriptional activator, a replication enhancer, and C4 protein in the complementary sense. DNA-B encodes a nuclear shuttling protein in the viral sense and a movement protein in the complementary sense. The genomes of monopartite begomoviruses resemble the DNA-A component of bipartite begomoviruses. 

Begomoviruses are considered one of the largest and most important groups of emerging plant viruses that infect a wide range of important vegetable, root, and fiber crops, mainly in tropical and subtropical regions. Although global losses caused by begomoviruses are difficult to estimate because of the vast number of crops they attack, they are certainly in the range of billions of dollars annually. One of the areas where begomoviruses are widely distributed, both in crops and wild plants, is Latin America. Leguminous crops, mainly common bean (*Phaseolus vulgaris*) and soybean (*Glycine max*), are frequently infected by begomoviruses in Latin America, having been reported in Argentina, Brazil, Colombia, Cuba, Dominican Republic, Ecuador, Guatemala, Mexico, Puerto Rico, and Venezuela [5,6,7,8,9,10,11,12,13,14,15,16,17]. In Venezuela, fifteen begomovirus species have been described [11,18,19,20,21,22,23,24,25,26,27,28,29,30], most of which affect tomato crops and wild plants. Only two of these begomoviruses, bean chlorosis virus (BCV) and bean white chlorosis mosaic virus (BWCMV), have been found in a leguminous plant species, common bean. Both BCV and BWCMV were each described as infecting a single plant [11], and no information on distribution and alternative hosts is available. Although a variety of symptoms resembling those caused by begomoviruses have been observed in leguminous plants in the last 20 years in Venezuela, to date, there have been limited efforts made to molecularly characterize the putative viruses causing them. 

Several DNA satellites, the well-known betasatellites [31,32] and alphasatellites [33,34] and the most recently recognized deltasatellites [21,35], have been associated with begomoviruses. These DNA satellites depend on helper begomoviruses for replication (except alphasatellites), encapsidation, movement in the plant, and transmission by insect vectors [36,37]. Alphasatellites, mainly associated with monopartite Old World begomoviruses, possess a genome that encodes a replication-associated protein needed for their replication. Betasatellites, associated with many monopartite Old World begomoviruses, are essential for the induction of typical disease symptoms. The βC1 protein encoded by the betasatellite genome has important roles in symptom induction and suppression of transcriptional and post-transcriptional gene silencing [38]. Unlike betasatellites and alphasatellites, deltasatellites lack coding capacity. All deltasatellites share several genomic features such as their size, which is about one-quarter of the begomovirus genome component, a stem-loop containing the conserved begomovirus nonanucleotide TAATATTAC, a putative secondary stem-loop structure located close to begomovirus iteron-like sequences, a short region with the sequence identity of the betasatellite conserved region, and an A-rich region [35]. The most well-characterized deltasatellites have been naturally associated with (i) a monopartite Old World begomovirus infecting tomato in Australia [39]; (ii) bipartite begomoviruses infecting three wild malvaceous plant species in Cuba [35]; (iii) monopartite Old World begomoviruses infecting sweet potato (*Ipomoea batatas*) and *I. indica* (sweepoviruses) in Spain and Portugal [21]; and (iv) a sweepovirus infecting *Merremia dissecta* in Venezuela [21]. In some cases, deltasatellites reduce or increase the accumulation of the helper begomovirus, but rarely modify the symptoms caused by them [40,41].

The aim of the present study was to investigate the presence of begomoviruses and associated DNA satellites infecting cultivated and wild leguminous plants in Venezuela. As a result, the presence of bipartite begomoviruses was shown in 37% of the collected samples from both crops and wild plants, with four begomoviruses constituting novel species. Additionally, a deltasatellite, constituting a novel species, was found infecting 15% of the collected samples. This is the first deltasatellite found in legumes and in a crop in the New World, and its dependence on the begomovirus cabbage leaf curl virus (CabLCV) for replication was shown experimentally.

## 2. Materials and Methods

### 2.1. Plant Sample Collection and Molecular Identification

**Figure 1:**
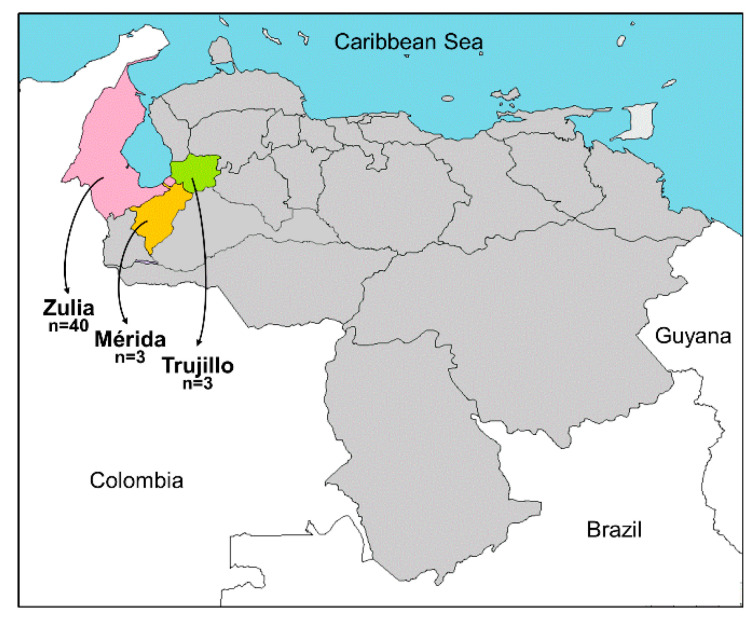
Map of Venezuela showing the geographic location of the states of Zulia (pink), Mérida (orange), and Trujillo (green). The number of samples (*n*) collected in each state is indicated.

**Figure 2:**
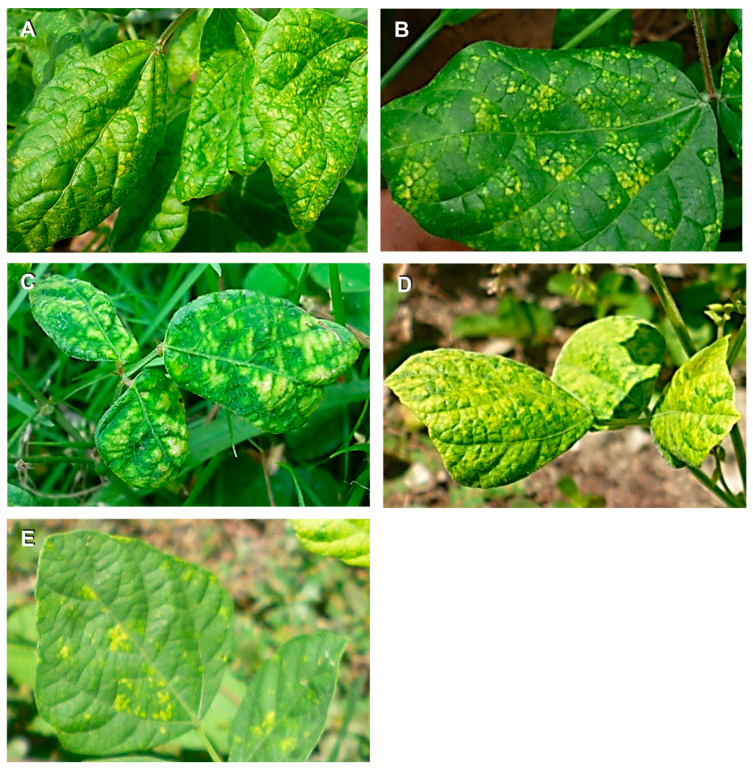
Photographs of some collected leguminous plants showing virus-like symptoms. Samples V3 (**A**, *Macroptilium bracteatum*), V4 (**B**, *M. bracteatum*), V7 (**C**, *Desmodium scorpiurus*), V9 (**D**, *Rhynchosia minima*), and V11 (**E**, *R. minima*).

A survey was conducted in important leguminous production areas of northwestern Venezuela (localities from the states of Zulia, Mérida, and Trujillo) from September–October 2017 (Figure 1). Forty-six leguminous (family Fabaceae, syn. Leguminosae) plants with virus-like symptoms such as yellow mosaic, leaf rugose, and leaf curl were collected. Plants included the economically important crops cowpea (*Vigna unguiculata*, *n* = 24), common bean (*Phaseolus vulgaris*, *n* = 4), black gram (*Vigna mungo*, *n* = 2), mung bean (*Vigna radiata*, *n* = 1), faba bean (*Vicia faba*, *n* = 1), and wild plants (*Rhynchosia minima*, *n* = 7; *Macroptilium bracteatum*, *n* = 3; *Desmodium scorpiurus*, *n* = 4) (Table 1, Figure 2). Morphological identification of the wild plants was molecularly confirmed by DNA barcoding using chloroplast *rbcL* and *matK* genes [42]. Leaf samples were dried using silica gel and maintained at room temperature in the dark until analysis.

### 2.2. DNA Extraction and Full-Length Begomovirus and Deltasatellite Genome Cloning from Field Samples

Total DNA was extracted from dried leaf tissue using a modified CTAB method [43] and was used as a template for rolling-circle amplification (RCA) using φ29 DNA polymerase (TempliPhi kit, GE Healthcare, Buckinghamshire, UK). Amplified RCA products were digested with a set of restriction enzymes (HpaII, which recognizes a 4-nt site, and BamHI, EcoRI, HindIII, NcoI, NheI, and SalI, which recognize 6-nt sites). Restriction products were analyzed in 1% agarose electrophoresis gels in Tris-acetate-EDTA buffer that were stained with ethidium bromide and visualized under UV light. The selected digested RCA products (~2.7 and ~0.7 kbp) corresponding to putative full-length begomoviral genome components and deltasatellite genomes, respectively, were cloned into pBlueScript II SK (+) (Stratagene, La Jolla, CA, USA). Recombinant plasmid DNA was transformed into *Escherichia coli* DH5α by electroporation, and selected clones were sequenced by Macrogen Inc. (Seoul, South Korea).

### 2.3. Sequence Analyses

The comparison of initial sequence similarity was performed using the BLAST program (https://blast.ncbi.nlm.nih.gov/Blast.cgi, accessed on 1 September 2021). Sequences were aligned with MUSCLE [44], and pairwise identity scores were calculated using SDT (sequence demarcation tool) [45]. DNA secondary structure prediction for the putative secondary stem-loop structure present in deltasatellites was performed by free energy minimization using the UNAfold web server (http://www.unafold.org/mfold/applications/dna-folding-form.php, accessed on 1 September 2021) [46,47]. MEGA 7 [48] was used for phylogenetic analysis using the neighbor-joining method [49] and the evolutionary distances were computed using the p-distance method [50].

### 2.4. Construction of Infectious Clones and Plant Agroinoculation

Begomovirus (DNA-A and DNA-B) and deltasatellite infectious clones were constructed from monomeric genomic components cloned from sample V9. Inserts from monomeric clones were released from the plasmids, re-ligated, and subjected to RCA. Each RCA product was partially digested to produce dimeric molecules that were cloned in a plasmid vector. The inserts of the dimeric clones were excised and subcloned in a binary vector. Ligation reactions were transformed in *Escherichia coli* DH5α by electroporation (25 μF, 200 Ω, 2500 V) in a Gene Pulser Xcell Electroporation System (Bio-Rad, Hercules, CA, USA). Clones were verified by digestion, and those with inserts of the expected size were sequenced at Macrogen Inc. (Seoul, South Korea). Head-to-tail dimeric constructs were transferred to *Agrobacterium tumefaciens* strain C58C1 by electroporation using the conditions described above. Details on restriction enzymes and vectors used for cloning are shown in Table S1.

For agroinoculation assays, *A. tumefaciens* cultures harboring each dimeric construct were added (1:1000) to YEP liquid media containing kanamycin (50 µg mL^−1^) and rifampicin (50 µg mL^−1^) and grown at 28 °C for two days. Cultures were centrifuged at 3100× *g* for 20 min at 4 °C. The cultures were decanted from the media, and the pellets were resuspended in *Agrobacterium* inoculation buffer (10 mM MES, 10 mM MgCl_2_, and 150 μM acetosyringone, pH 5.6). The optical density was adjusted to 1 at 600 nm. *Nicotiana benthamiana* plants at the four-leaf stage were inoculated with *A. tumefaciens* cultures by stem puncture inoculation. Plants were maintained in an insect-free growth chamber (25 °C during the day and 18 °C at night, 70% relative humidity, with a 16-h photoperiod at 250 µmol s^−1^ m^−2^ of photosynthetically active radiation) for further analysis.

### 2.5. Begomovirus and Deltasatellite Detection

Begomovirus genome components (DNA-A and DNA-B) and deltasatellite genomes were detected in agroinoculated plants by molecular hybridization. At 38 days post-inoculation (dpi), the apical leaves were used for tissue blots of petiole cross-sections (tissue printing) performed on positively charged nylon membranes. Hybridization of the membranes was carried out using specific digoxigenin-labelled DNA probes. Probes were synthesized by PCR according to the DIG-labelling detection kit (Roche Diagnostics, Mannheim, Germany) with primers and amplification programs shown in Table S2. Hybridization was carried out under high stringency conditions [washing steps at 65 °C in 0.19× SSC (15 mM NaCl and 1.5 mM sodium citrate) and 0.1% sodium dodecyl sulfate] following standard procedures. Membranes were treated with CDP-Star (Roche Diagnostic, Mannheim, Germany), and hybridization signals were detected on X-ray film (Kodak, Rochester, NY, USA) following a conventional photographic process. Cabbage leaf curl virus detection in field-infected plants was also carried out by PCR using specific primers designed to synthesize the above-mentioned probes.

## 3. Results and Discussion

### 3.1. Known and Novel New World Begomoviruses Infect Leguminous Plants in Venezuela 

Analysis of leaf samples by digestion of RCA products with several restriction enzymes and PCR revealed that 37% (17 of 46) of the plants sampled were infected by begomoviruses (samples V1–V4, V6–V13, V20–V23, and V35) (Table 1). Full-length viral genomes (DNA-A and DNA-B) were cloned and sequenced from cultivated (samples V2 and V35 [cowpea] and V35 [black gram]) and wild plants (samples V3 and V4 [*Macroptilium bracteatum*], V6 and V7 [*Desmodium scorpiurus*], and V9–V11, V20–V23 [*Rhynchosia minima*]). These samples were collected in the states of Zulia (three municipalities) and Mérida (one municipality) (Table 1). GenBank accession numbers of full-length begomovirus genome components (DNA-A and DNA-B) isolated in this work are listed at the end of the manuscript and included in Table S3.

Eleven samples (three cowpea [V1 and V2], two black gram [V12 and V13], one *D. scorpiurus* [V8], and five *R. minima* [V9, V10, V20, V22, and V23]) were infected by CabLCV, a typical New World begomovirus (Table 1). The complete genome sequences of DNA-A and DNA-B of CabLCV from eight infected plants from different hosts were sequenced after RCA and cloning (samples V2 and V35 from cowpea, V13 from black gram and V9, V10, V20, V22, and V23 from *R. minima*). CabLCV isolates showed a high sequence identity (96.5–100% for DNA-A and 93.6–100% for DNA-B). These isolates were also closely related (96.6–97.6%) to the isolate of CabLCV infecting common bean (MH359390) and *R. minima* (MH359394) in Ecuador [12]. In samples V1 (cowpea), V8 (*D. scorpiurus*), and V12 (black gram), CabLCV was detected by PCR with specific primers (Table S2) for the DNA-A and DNA-B components. Although CabLCV was first found to infect cabbage in the United States [51], the virus was found later infecting wild plants of the family Fabaceae in Mexico (*Desmodium* sp. and *Rhynchosia* sp.) [52], Jamaica (*Rhynchosia* sp.) (KP641347-KP641350), and Ecuador (*Mucuna pruriens*) [12]. Recently, CabLCV has also been found to infect leguminous crops including soybean in Cuba and common bean, cowpea, and pigeon pea in Ecuador [12]. These findings, along with those reported here, highlight the importance of the presence of CabLCV in legumes. Begomoviruses isolated from the other infected plants were different from CabLCV, although they also showed the typical genome organization of bipartite New World begomoviruses.

DNA-A isolated from *R. minima* samples V11 and V21 were 99% identical and showed the highest sequence identity (87.2–87.5%) with an isolate of *Rhynchosia golden mosaic virus* (RhGMV, EU339938) infecting *R. minima* in Mexico. DNA-B from the same samples showed an identity of 98.5%, and the highest sequence identity (80.2%) was with a RhGMV isolate (DQ356429) infecting soybean in Mexico. Interestingly, the two plants infected by this virus were sampled in different municipalities of the state of Zulia, Maracaibo, and Sucre, separated by about 180 km by Lake Maracaibo. This suggests that this virus is probably spread, at least in other municipalities of the state of Zulia, that were not surveyed.

DNA-A from an *M. bracteatum* sample (V3) showed the highest identity (81.6%) with an isolate of CabLCV (KP641349) infecting *R. minima* in Jamaica. DNA-B from the same sample showed the highest identity (74.3%) with an isolate of bean golden mosaic virus (BGYMV, L01636) infecting common bean in the Dominican Republic [14].

DNA-A from another *M. bracteatum* sample (V4) showed the highest identity (91.3%) with bean leaf crumple virus (KX857725) infecting common bean in Colombia [7]. However, DNA-B from the same sample was most closely related (74.5%) to BGYMV (L01636) [14].

DNA-A from sample V6 of *D. scorpiurus* showed the highest identity (82.3%) with an isolate of tomato yellow mottle virus (KC176780) from Costa Rica. DNA-B from the same sample was most closely related (73.2%) to bean latent virus (BLV, MN158326) from Mexico [16].

DNA-A and DNA-B from sample V7 of *D. scorpiurus* showed the highest identity (82 and 73%, respectively) with two isolates of CabLCV (KT192632 and KT381194) infecting soybean in Cuba.

The current begomovirus species demarcation criteria established that a new isolate should be considered to belong to a novel species if the highest percentage of pairwise identity (full-length genome for monopartite begomoviruses or DNA-A component for bipartite begomoviruses) with known begomoviruses is <91%. Thus, begomoviruses isolated from samples V11 and V21, V3, V6, and V7 would constitute novel species and the following names are proposed: *Rhynchosia mottle virus* (RhMoV, isolates VE-Rh V11–17 and VE-Rh V21–17 from samples V11 and V21, respectively), *Macroptilium mottle virus* (MacMoV, isolate VE-Mac V3–17 from sample V3), *Desmodium mosaic virus* (DesMV, isolate VE-Des V6–17, sample V6), and *Desmodium yellow spot virus* (DesYSV, isolate VE-Des V7–17, from sample V7).

DNA-A pairwise identities of 94% is the current demarcation threshold for begomovirus strains. Thus, the begomovirus from sample V4 would constitute a new strain of *Bean leaf crumple virus* (BLCrV-VE [VE-Mac V4–17]) as its DNA-A was 91.3% identical to the most closely related DNA-A (KX857725).

**Figure 3:**
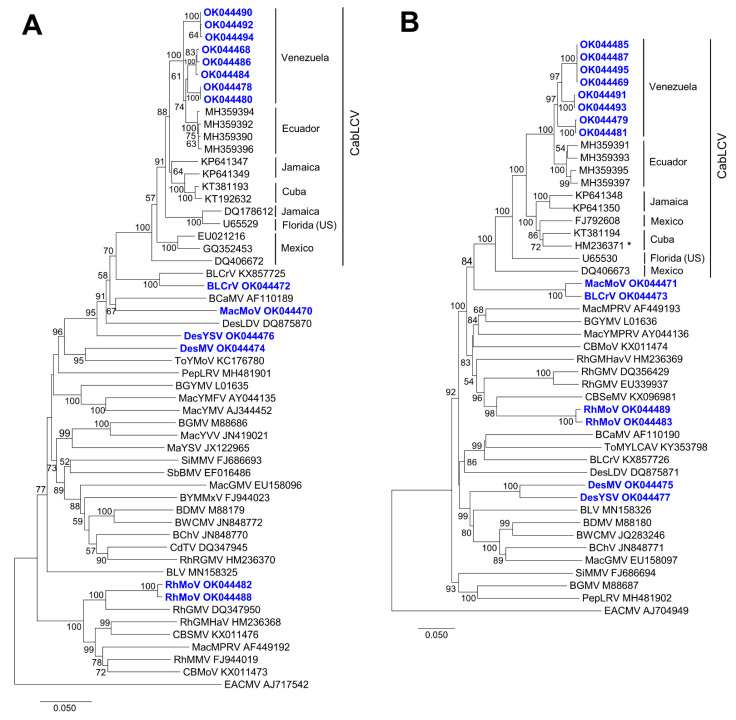
Phylogenetic tree illustrating the relationship of DNA-A (**A**) and DNA-B (**B**) from begomoviruses described in this work (BLCrV, bean leaf crumple virus; CabLCV, cabbage leaf curl virus; DesMV, Desmodium mosaic virus; DesYSV, Desmodium yellow spot virus; MacMoV, Macroptilium mottle virus; RhMoV, Rhynchosia mottle virus) to other New World begomoviruses. The tree was constructed by the neighbor-joining method (1000 replicates) with the MEGA 7 program [48]. GenBank accession numbers are shown in the tree and the names of the viruses follow: BCaMV, bean calico mosaic virus; BChV, bean chlorosis virus; BDMV, bean dwarf mosaic virus; BGMV, bean golden mosaic virus; BGYMV, bean golden yellow mosaic virus; BLCrV, bean leaf crumple virus; BLV, bean latent virus; BWCMV, bean white chlorosis mosaic virus; BYMMxV, bean yellow mosaic Mexico virus; CBMoV, common bean mottle virus; CBSMV, common bean severe mosaic virus; CdTV, chino del tomate virus; DesLDV, Desmodium leaf distortion virus; MacGMV, Macroptilium golden mosaic virus; MacMPRV, Macroptilium mosaic Puerto Rico virus; MacYMFV, Macroptilium yellow mosaic Florida virus; MacYMV, Macroptilium yellow mosaic virus; MacYSV, Macroptilium yellow spot virus; MacYVV, Macroptilium yellow vein virus; PepLRV, pepper leafroll virus; RhGMHaV, Rhynchosia golden mosaic Havana virus; RhGMV, Rhynchosia golden mosaic virus; RhMMV, Rhynchosia mild mosaic virus; RhRGMV, Rhynchosia rugose golden mosaic virus; SbBMV, soybean blistering mosaic virus; SiMMV, Sida micrantha mosaic virus; ToYMoV, tomato yellow mottle virus. * HM236371 corresponds to DNA-B of RhRGMV. The OW begomovirus East African cassava mosaic virus (EACMV) was used as an outgroup. Bootstrap values are shown for supported branches (>50%). The bars below the trees indicate the number of nucleotide substitutions per site.

The analysis of phylogenetic relationships of the DNA-A and DNA-B genomes described in this work with isolates of other begomoviruses native to the New World infecting leguminous plants showed that the novel begomoviruses belong to different lineages. CabLCV isolates are included in a major clade with high bootstrap support (100%). Within this clade, a minor clade includes isolates from Venezuela (described in this work) and Ecuador [12], all of which were found to infect plants of the family Fabaceae (Figure 3). 

DNA-Bs of Macroptilium mottle virus and bean leaf crumple virus were grouped in sister branches, as occurs with DNA-Bs of DesMV and DesYSV. However, their DNA-As were not grouped together. This represents an additional example of the dissimilar evolutionary history undergone by both begomovirus genome components [53]. The same happens, for example, with Rhynchosia rugose golden mosaic virus (RhRGMV); DNA-A is phylogenetically related to viral isolates infecting bean, soybean, and *Wissadula amplissima* (Figure 3A), while DNA-B is included in the clade that contains all the CabLCV isolates (Figure 3B).

Of all 46 symptomatic plants, only 17 were shown to be infected by begomoviruses. This could be explained because unspecific symptoms could be caused by RNA viruses (not analyzed here), other pathogens, or even nutritional deficiencies. The identification of novel begomovirus species in legumes stressed the importance of these viruses as a limiting factor in the production of these crops in Central and South America and the Caribbean. Additionally, the presence of begomoviruses in cultivated and wild legumes suggests a reservoir role for the non-cultivated plants in the emergence of viruses that could be a threat to nearby crops. The role of wild plants in begomovirus emergence has been proposed in the case of numerous begomoviruses infecting, for example, tomato crops [54,55,56,57,58,59].

### 3.2. Deltasatellites Belonging to a Novel Species Are Associated with CabLCV Infecting Wild and Cultivated Leguminous Plants 

**Figure 4:**
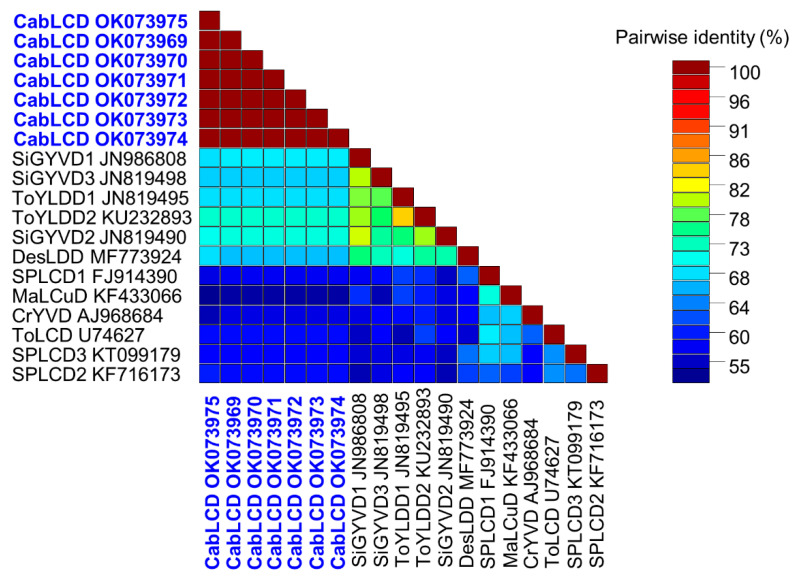
Color-coded matrix of pairwise nucleotide sequence identity of isolates of cabbage leaf curl deltasatellite (CabLCD) (bold in blue) described in this work with representative isolates of other deltasatellite species (CrYVD, Croton yellow vein deltasatellite; DesLDD, Desmodium leaf distortion deltasatellite; MaLCuD, Malvastrum leaf curl deltasatellite; SiGYVD1, Sida golden yellow vein deltasatellite 1; SiGYVD2, Sida golden yellow vein deltasatellite 2; SiGYVD3, Sida golden yellow vein deltasatellite 3; SPLCD1, sweet potato leaf curl deltasatellite 1; SPLCD2, sweet potato leaf curl deltasatellite 2; SPLCD3, sweet potato leaf curl deltasatellite 3; ToLCD, tomato leaf curl deltasatellite; ToYLDD1, tomato yellow leaf distortion deltasatellite 1; ToYLDD2, tomato yellow leaf distortion deltasatellite 2).

Deltasatellites were found to infect seven of the 11 leguminous plants infected by CabLCV including cowpea (samples V1 and V2), black gram (V12 and V13), *D. scorpiurus* (V8), and *R. minima* (V9 and V10) (Table 1). GenBank accession numbers of full-length deltasatellite genomes isolated in this work are listed at the end of the manuscript and included in Table S3. These plants were collected in two municipalities in the state of Zulia and one municipality in the state of Mérida. Deltasatellite isolates showed identities of 100% between them and the highest identity (73.9%) with a previously characterized deltasatellite, tomato yellow leaf distortion deltasatellite 2 (KU232893), associated with the bipartite begomovirus tomato yellow leaf distortion virus isolated from *Sidastrum micranthum* in Cuba [40] (Figure 4). The current threshold for species demarcation in the genus *Deltasatellite* is 91% nucleotide sequence identity when considering full-length sequences. Thus, the deltasatellite described here would constitute a novel species. Considering the association of this deltasatellite with CabLCV, the name *Cabbage leaf curl deltasatellite* is proposed for this species.

**Figure 5:**
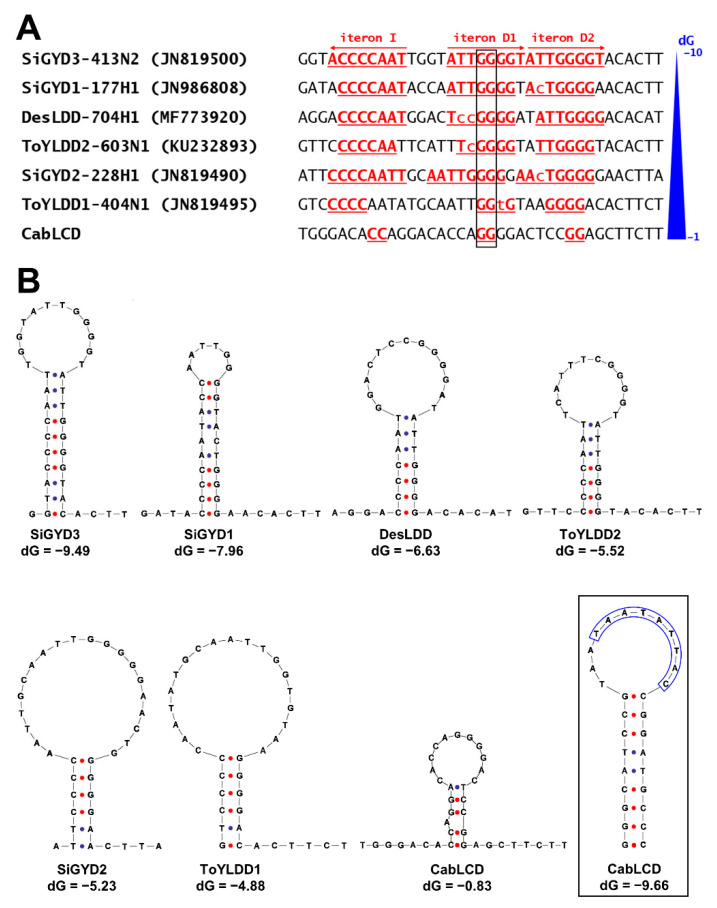
In silico analysis of the secondary stem-loop structure present in deltasatellites. (**A**) Alignment of the iteron-like sequences of the deltasatellite characterized in this work (cabbage leaf curl deltasatellite, CabLCD) and other New World deltasatellites (Sida golden yellow deltasatellite 3, SiGYD3; Sida golden yellow deltasatellite 1, SiGYD1; Desmodium leaf distortion deltasatellite, DesLDD; tomato yellow leaf distortion deltasatellite 2, ToYLDD2; Sida golden yellow deltasatellite 2, SiGYD2; tomato yellow leaf distortion deltasatellite 1, ToYLDD1). Direct (D1 and D2) and inverse (I) iterons are underlined and in red. Non-conserved nucleotides present in imperfect iterons are in lower case. The conserved GG motif in iteron D1 is boxed. Deltasatellites are ordered from lower to higher Gibbs free energy (dG) of the predicted secondary structure for the genomic region shown. (**B**) Secondary structures for the putative secondary stem-loop structure present in CabLCD and the other deltasatellites included in panel A predicted by free energy minimization. The CabLCD stem-loop containing the conserved nonanucleotide TAATATTAC (boxed and in blue) is included for comparison.

The cloned and sequenced deltasatellites, one per sample, were 666 nt long and contained typical features found in these subviral agents (i.e., a stem-loop containing the conserved nonanucleotide TAATATTAC, a short region with sequence identity with the betasatellite-conserved region, and an A-rich region) [35]. The secondary stem-loop located close to begomovirus iteron-like sequences, which are also typical of deltasatellites, was not evident. However, a more detailed analysis localized the iteron-like sequences of the deltasatellites, which were characterized here after successful alignment with the corresponding region of other New World deltasatellites (Figure 5A). These sequences were associated with the secondary stem-loop in previously characterized deltasatellites [35,39]. Figure 5B shows the predicted secondary structures for the genomic region where the iteron-like sequences are located, both in the deltasatellites characterized in this work and the other New World deltasatellites analyzed in Figure 5A. These analyses showed a gradation among New World deltasatellites, both for the length of the iteron-like sequences and the Gibbs free energy (dG) of the predicted secondary stem-loops, with a high negative correlation between both parameters (R^2^ = 0.7108) (Figure S1). This suggests that the predicted secondary stem-loop, although it seems to be a universal feature in deltasatellites, presents a significant variation in the level of structuration, which could be related to the presence/absence of a specific function.

**Figure 6:**
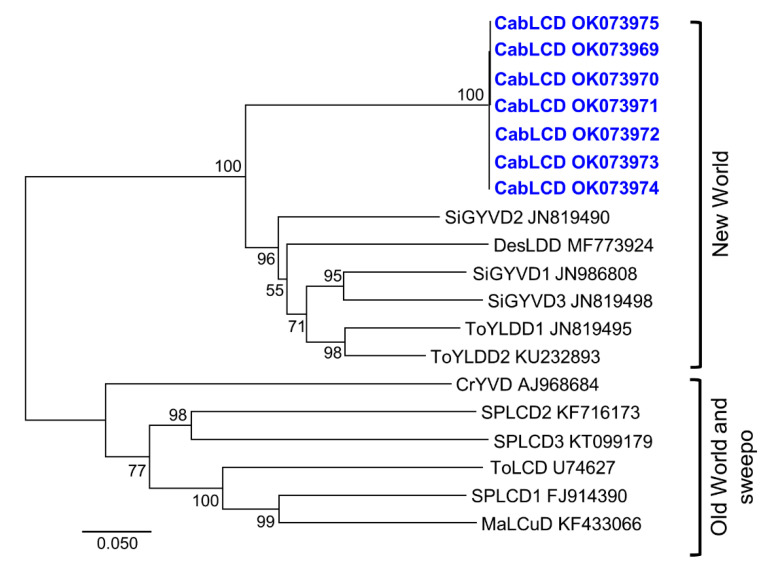
Phylogenetic tree illustrating the relationships of isolates of cabbage leaf curl deltasatellite (CabLCD) (bold in blue) described in this work with representative isolates of other deltasatellite species. The tree was constructed by the neighbor-joining method (1000 replicates) with MEGA 7 [48]. The bar below the tree indicates the number of nucleotide substitutions per site. Major deltasatellite clusters associated with bipartite New World begomoviruses (New World) and monopartite Old World begomoviruses and sweepoviruses (Old World and sweepo) are indicated. Deltasatellite names are in the Figure 4 caption.

Phylogenetic analysis including one isolate of each deltasatellite species recognized to date placed cabbage leaf curl deltasatellite (CabLCD) within the cluster that includes New World deltasatellites (Figure 6). CabLCD is the first deltasatellite found to infect legumes and was the first found in the New World to infect a crop plant. Prior to this work, deltasatellites had been found in the New World in wild plants associated with the New World begomoviruses Sida golden yellow vein virus, tomato yellow leaf distortion virus, and Desmodium leaf distortion virus infecting malvaceous plants (*Sida micrantha*, *Malvastrum coromandelianum,* and *Corchorus siliquosus*) in Cuba [35] and the sweepovirus sweet potato leaf curl virus infecting the convolvulaceous *Merremia dissecta* in Venezuela [21]. Deltasatellites have also been detected in the Americas in whiteflies using vector-enabled metagenomic approaches in Puerto Rico [60] and Florida (United States) [61]. However, in the Old World, some deltasatellites have been found in economically important crops: ToLCD in tomato in Australia [39] and SPLCD1 in sweet potato in Spain [21]. 

The presence of a deltasatellite infecting plants of the family Fabaceae suggests that these subviral agents are probably capable of infecting members of additional plant families and are associated in nature with a wider range of helper begomoviruses than previously assumed.

### 3.3. Cabbage Leaf Curl Virus Acts as a Helper Virus for CabLCD

**Figure 7:**
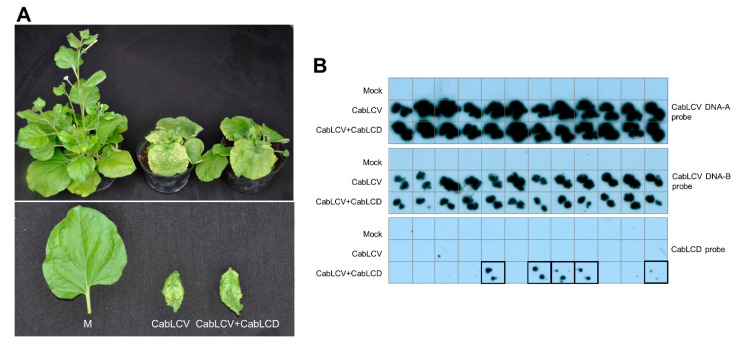
Agroinoculation experiments with cabbage leaf curl virus and cabbage leaf curl deltasatellite in *Nicotiana benthamiana*. (**A**) Symptoms caused by CabLCV and CabLCVD on agroinoculated *N. benthamiana* plants. Mock-inoculated controls (M) are shown on the left. (**B**) CabLCV (DNA-A and DNA-B) and CabLCD were detected by molecular hybridization after tissue printing on nylon membranes using specific digoxigenin-labelled DNA probes for each viral/subviral component. Prints of mock-inoculated plants were included as negative controls. Prints of CabLCD-infected plants are outlined.

*Nicotiana benthamiana* plants agroinoculated with CabLCV alone or in the presence of CabLCD were analyzed by tissue printing hybridization of apical leaves. In two independent experiments, all CabLCV and CabLCV/CabLCD inoculated plants became symptomatic, showing severe stunting, leaf deformation, and mild leaf yellowing (Figure 7A). Tissue print hybridization with a probe specific for each genome component of CabLCV confirmed the presence of both DNA-A and DNA-B in all inoculated plants (Figure 7B). In contrast, CabLCD was detected in 41.7 and 25.0% of the inoculated plants in the two independent experiments where virus/deltasatellite co-inoculation was carried out (Table 2). No differences in symptomatology were observed in the CabLCV-infected plants in the presence or absence of CabLCD (Figure 7B). The inability of deltasatellites to influence the symptomatology caused by the helper begomoviruses has been observed in most cases experimentally analyzed to date. Known examples are the New World Sida golden yellow vein virus (SiGYVV)/Sida golden yellow vein deltasatellite 1 (SiGYVD1) and tomato yellow leaf distortion virus/tomato yellow leaf distortion deltasatellite 2 (ToYLDD2) both in the natural malvaceous host plants and the experimental host *N. benthamiana*, and tomato leaf deformation virus (ToLDeV)/SiGYVD1 and ToLDeV/ToYLDD2 in *N. benthamiana* [40]. The same has been observed in coinfections of sweet potato leaf curl deltasatellite 1 (SPLCD1) and the bipartite Old World begomovirus tomato leaf curl New Delhi virus (ToLCNDV), the bipartite New World begomovirus SiGYVV, the monopartite New World begomovirus ToYLDeV or the curtovirus beet curly top virus in *N. benthamiana*; SPLCD1 and ToLCNDV in zucchini; and SPLCD1 and the sweepovirus sweet potato leaf curl virus (SPLCV) in most natural host plants [41]. Exceptions include the slight decrease in symptomatology of SPLCD1 and SPLCV observed in *Ipomoea setosa* and *I. nil* and SPLCD1 and tomato yellow leaf curl virus or tomato yellow leaf curl Sardinia virus in *N. benthamiana* and tomato.

## 4. Conclusions

In this study, six bipartite begomoviruses were found to be associated with symptomatic leguminous plants, both cultivated and wild, in Venezuela. Interestingly, four of the begomoviruses reported here constitute novel species. CabLCV, a begomovirus previously described in other countries from the Americas, was the most widespread in the analyzed samples. This is the first report of CabLCV in Venezuela and the first time that this virus has been found infecting black gram and *Desmodium scorpiurus*. In addition, a novel deltasatellite species was described associated with CabLCV, *Cabbage leaf curl deltasatellite*. This is the first time that a deltasatellite has been found infecting a species of the Fabaceae family as well as economically important crops in the Americas. The results obtained in this study contribute to the explanation of the causes of symptoms that have been observed in leguminous plants in the last 20 years in Venezuela. Understanding the actual prevalence of begomovirus–deltasatellite complexes is fundamental to the choice and improvement of control methods to prevent or eliminate the viruses that cause them, mainly in those countries where they are understudied.

## Figures and Tables

**Table 1:** Plant host, place of collection, and begomovirus/deltasatellite content of the samples analyzed in this study.

Plant Species	State	Municipality	Sample Code	Begomovirus	Deltasatellite
Black gram	Mérida	Tulio Febres-Cordero	V12	CabLCV *	CabLCD
	Mérida	Tulio Febres-Cordero	V13	CabLCV	CabLCD
Common bean	Trujillo	Escuque	V14	-	-
	Trujillo	Escuque	V15	-	-
	Trujillo	Escuque	V16	-	-
	Mérida	Justo Briceño	V19	-	-
Cowpea	Zulia	Jesús Enrique Lossada	V1	CabLCV *	CabLCD
	Zulia	Jesús Enrique Lossada	V2	CabLCV	CabLCD
	Zulia	Jesús Enrique Lossada	V24	-	-
	Zulia	Jesús Enrique Lossada	V26	-	-
	Zulia	Jesús Enrique Lossada	V27	-	-
	Zulia	Jesús Enrique Lossada	V28	-	-
	Zulia	Jesús Enrique Lossada	V29	-	-
	Zulia	Jesús Enrique Lossada	V30	-	-
	Zulia	Jesús Enrique Lossada	V31	-	-
	Zulia	Jesús Enrique Lossada	V32	-	-
	Zulia	Jesús Enrique Lossada	V33	-	-
	Zulia	Jesús Enrique Lossada	V34	-	-
	Zulia	Jesús Enrique Lossada	V35	CabLCV	-
	Zulia	Jesús Enrique Lossada	V36	-	-
	Zulia	Jesús Enrique Lossada	V37	-	-
	Zulia	Jesús Enrique Lossada	V38	-	-
	Zulia	Jesús Enrique Lossada	V39	-	-
	Zulia	Jesús Enrique Lossada	V40	-	-
	Zulia	Jesús Enrique Lossada	V41	-	-
	Zulia	Jesús Enrique Lossada	V42	-	-
	Zulia	Jesús Enrique Lossada	V43	-	-
	Zulia	Jesús Enrique Lossada	V44	-	-
	Zulia	Jesús Enrique Lossada	V45	-	-
	Zulia	Jesús Enrique Lossada	V46	-	-
Faba bean	Zulia	Sucre	V18	-	-
Mung bean	Zulia	Sucre	V17	-	-
*Desmodium scorpiurus*	Zulia	Sucre	V6	DesMV	-
	Zulia	Sucre	V7	DesYSV	-
	Zulia	Sucre	V8	CabLCV *	CabLCD
	Zulia	Maracaibo	V47	-	-
*Macroptilium bracteatum*	Zulia	Jesús Enrique Lossada	V3	MacMoV	-
	Zulia	Jesús Enrique Lossada	V4	BLCrV	-
	Zulia	Jesús Enrique Lossada	V5	-	-
*Rhynchosia minima*	Zulia	Sucre	V9	CabLCV	CabLCD
	Zulia	Sucre	V10	CabLCV	CabLCD
	Zulia	Sucre	V11	RhMoV	-
	Zulia	Sucre	V20	CabLCV	-
	Zulia	Maracaibo	V21	RhMoV	-
	Zulia	Maracaibo	V22	CabLCV	-
	Zulia	Maracaibo	V23	CabLCV	-

For all begomovirus and deltasatellite isolates identified, full-length components were obtained, except for those sequences marked with an asterisk (*) for which only a partial DNA-A sequence was obtained. BLCrV, bean leaf crumple virus; CabLCV, cabbage leaf curl virus; DesMV, Desmodium mosaic virus; DesYSV, Desmodium yellow spot virus; MacMoV, Macroptilium mottle virus; RhMoV, Rhynchosia mottle virus; CabLCD, cabbage leaf curl deltasatellite.

**Table 2:** Infectivity of cabbage leaf curl virus (CabLCV) and the associated cabbage leaf curl deltasatellite (CabLCD) in *Nicotiana benthamiana* plants.

Virus/Deltasatellite	No. Infected Plants/No. Agroinoculated Plants			
	Experiment 1		Experiment 2	
	CabLCV	CabLCD	CabCV	CabLCD
CabLCV	12/12	-	12/12	-
CabLCV + CabLCD	12/12	5/12	12/12	3/12

## Data Availability

Nucleotide sequences obtained in this study have been deposited in GenBank under accession numbers OK044468–OK044495 and OK073969–OK073975.

## Supplementary Materials

The following are available online at https://www.mdpi.com/article/10.3390/biology10111125/s1: Figure S1: Correlation analysis between the length of the iteron-like sequences and the Gibbs free energy (dG) of the predicted secondary stem-loops of New World deltasatellites; Table S1: Cloning strategy to obtain the infectious dimer plasmids of cabbage leaf curl virus (CabLCV) DNA-A and DNA-B and cabbage leaf curl deltasatellite (CabLCD); Table S2: Primers and PCR conditions employed to prepare the DNA probes used in this work; Table S3: GenBank accession numbers of full-length begomovirus (DNA-A and DNA-B components) and deltasatellite genomes characterized in this work.
