# Supplementary material for: Insights into Emerging Begomovirus–Deltasatellite Complex Diversity: The First Deltasatellite Infecting Legumes
Source: Biology (Basel). 2021 Nov 2;10(11):1125. doi: 10.3390/biology10111125 (PMC8615175; doi:10.3390/biology10111125)
Supplement: Supplementary file 1 [file biology-10-01125-s001.zip › biology-1426350-supplementary.pdf]

Insights into emerging begomovirus-deltasatellite complex diversity: the first deltasatellite infecting legumes

Elvira Fiallo-Olivé, Liseth Basstidas, Dorys T. Chirinos and Jesús Navas-Castillo

Supplementary Materials

Figure S1. Correlation analysis between the length of the iteron-like sequences and the Gibbs free energy (dG) of the predicted secondary stem-loops of New World deltasatellites.

Table S1. Cloning strategy to obtain the infectious dimer plasmids of cabbage leaf curl virus (CabLCV) DNA-A and DNA-B and cabbage leaf curl deltasatellite (CabLCD) genomes.

Table S2. Primers and PCR conditions employed to prepare the DNA probes used in this work.

Table S3. GenBank accession numbers of full-length begomovirus (DNA-A and DNA-B components) and deltasatellite genomes characterized in this work.

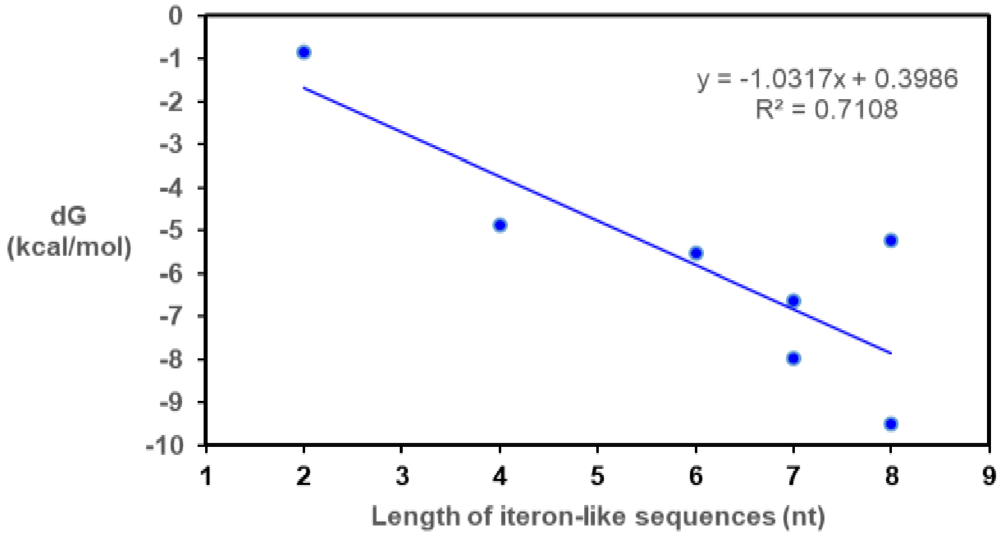

Figure S1. Correlation analysis between the length of the iteron-like sequences and the Gibbs free energy (dG) of the predicted secondary stem-loops of New World deltasatellites.

Table S1. Cloning strategy to obtain the infectious dimer plasmids of cabbage leaf curl virus (CabLCV) DNA-A and DNA-B and cabbage leaf curl deltasatellite (CabLCD) genomes.

DNA component/sample	Monomer vector/enzyme[1]	Enzyme used to obtain the dimeric molecule after RCA	Vector/enzyme used to clone the dimer	Enzymes used to subclone the dimer in the binary vector pCAMBIA0380	Infectious dimer plasmid
CabLCV DNA-A/V9	CabLCV DNA-A/SacI	SspI	pBSK(+)/EcoRV	EcoRI/SalI	pC0380dimCabLCV-A
CabLCV DNA-B/V9	CabLCV DNA-B/SacI	SacI	pUC18(+)/SacI	EcoRI/PstI	pC0380dimCabLCV-B
CabLCD DNA/V9	CabLCD DNA/EcoRI	EcoRI	pBSK(+)/EcoRI	BamHI/HindIII	pC0380dimCabLCD

[1]Enzyme used to clone the virus/satellite monomers and also used for digestion of monomeric clone to carry out RCA.

Table S2. Primers and PCR conditions employed to prepare the DNA probes used in this work.

Target virus/deltasatellite	Fragment size (bp)	Primers (5′-3′ sequence)	PCR program
CabLCV DNA-A	604	MA2671 (CACCAAGTTGTGGGCCTATATAACG)<br>MA2672 (CGTCACCTTTGCGTAGAACTTGTG)	3 min 95°C<br>x34 (45 s 95°C, 45 s 62.7°C, 45 s 72°C)<br>5 min 72°C
CabLCV DNA-B	492	MA2673 (GTTGCACGTAATCATGGTTCAAAGC)<br>MA2674 (CACACGCTTCAAAACATGGCGAAC)	3 min 95°C<br>x34 (45 s 95°C, 45 s 60.9°C, 45 s 72°C)<br>5 min 72°C
CabLCD	340	MA2675 (GTCTCATGATCAACTGTTCATGAAG)<br>MA2676 (CTAAGGCTGTGCCTAGGGAAATAC)	3 min 95°C<br>x34 (45 s 95°C, 45 s 59.7°C, 45 s 72°C)<br>5 min 72°C

Table S3. GenBank accession numbers of full-length begomovirus (DNA-A and DNA-B components) and deltasatellite genomes characterized in this work.

Virus/deltasatellite	Sample	Genome component	GenBank accession number
Cabbage leaf curl virus	V2	DNA-A	OK044468
		DNA-B	OK044469
	V9	DNA-A	OK044478
		DNA-B	OK044479
	V10	DNA-A	OK044480
		DNA-B	OK044481
	V13	DNA-A	OK044484
		DNA-B	OK044485
	V20	DNA-A	OK044486
		DNA-B	OK044487
	V22	DNA-A	OK044490
		DNA-B	OK044491
	V23	DNA-A	OK044492
		DNA-B	OK044493
	V35	DNA-A	OK044494
		DNA-B	OK044495
Rhynchosia mottle virus	V11	DNA-A	OK044482
		DNA-B	OK044483
	V21	DNA-A	OK044488
		DNA-B	OK044489
Macroptilium mottle virus	V3	DNA-A	OK044470
		DNA-B	OK044471
Bean leaf crumple virus	V4	DNA-A	OK044472
		DNA-B	OK044473
Desmodium mosaic virus	V6	DNA-A	OK044474
		DNA-B	OK044475
Desmodium yellow spot virus	V7	DNA-A	OK044476
		DNA-B	OK044477
Cabbage leaf curl deltasatellite	V1	DNA	OK073969
	V2	DNA	OK073970
	V8	DNA	OK073971
	V9	DNA	OK073972
	V10	DNA	OK073973
	V12	DNA	OK073974
	V13	DNA	OK073975
